# Nodulin 41, a novel late nodulin of common bean with peptidase activity

**DOI:** 10.1186/1471-2229-11-134

**Published:** 2011-10-10

**Authors:** Juan Elías Olivares, Claudia Díaz-Camino, Georgina Estrada-Navarrete, Xochitl Alvarado-Affantranger, Margarita Rodríguez-Kessler, Fernando Z Zamudio, Timoteo Olamendi-Portugal, Yamile Márquez, Luis Eduardo Servín, Federico Sánchez

**Affiliations:** 1 Departamento de Biología Molecular de Plantas, Instituto de Biotecnología/Universidad Nacional Autónoma de México, Av. Universidad 2001, Cuernavaca, Morelos, 62210, México; 2 Departamento de Medicina Molecular y Bioprocesos, Instituto de Biotecnología/Universidad Nacional Autónoma de México, Av. Universidad 2001, Cuernavaca, Morelos, 62210, México

## Abstract

**Background:**

The legume-rhizobium symbiosis requires the formation of root nodules, specialized organs where the nitrogen fixation process takes place. Nodule development is accompanied by the induction of specific plant genes, referred to as nodulin genes. Important roles in processes such as morphogenesis and metabolism have been assigned to nodulins during the legume-rhizobium symbiosis.

**Results:**

Here we report the purification and biochemical characterization of a novel nodulin from common bean (*Phaseolus vulgaris *L.) root nodules. This protein, called nodulin 41 (PvNod41) was purified through affinity chromatography and was partially sequenced. A genomic clone was then isolated via PCR amplification. PvNod41 is an atypical aspartyl peptidase of the A1B subfamily with an optimal hydrolytic activity at pH 4.5. We demonstrate that PvNod41 has limited peptidase activity against casein and is partially inhibited by pepstatin A. A PvNod41-specific antiserum was used to assess the expression pattern of this protein in different plant organs and throughout root nodule development, revealing that PvNod41 is found only in bean root nodules and is confined to uninfected cells.

**Conclusions:**

To date, only a small number of atypical aspartyl peptidases have been characterized in plants. Their particular spatial and temporal expression patterns along with their unique enzymatic properties imply a high degree of functional specialization. Indeed, PvNod41 is closely related to CDR1, an *Arabidopsis thaliana *extracellular aspartyl protease involved in defense against bacterial pathogens. PvNod41's biochemical properties and specific cell-type localization, in uninfected cells of the common bean root nodule, strongly suggest that this aspartyl peptidase has a key role in plant defense during the symbiotic interaction.

## Background

Leguminous plants can establish mutually beneficial associations with soil N_2_-fixing bacteria, mainly belonging to the Rhizobiacea family (rhizobia) [1,2]. This remarkable biological process culminates in the formation of specialized organs, the symbiotic nodules, where the N_2 _fixation process takes place. The legume-rhizobium interaction initiates with an exchange of molecular signals, a chemical dialog that leads to mutual recognition, the attachment of the bacteria to the plant root hairs, and the formation of the nodule meristem. Rhizobia invade plant roots via an infection thread made of plant material while a nodule primordium is simultaneously induced in the root cortex. Bacteria are released from infection threads into the cytoplasm of primordium cells by endocytosis and become surrounded by a plant-derived membrane, the peribacteroid membrane (PBM). The PBM is a physical and dynamic barrier between rhizobia and the cell's cytoplasm. Inside the hosting cell, the bacteria multiply, undergo a dramatic differentiation process including extreme cell enlargement, and finally become specialized N_2_-fixing bacteroids [3]. In fully developed bean nodules, two major tissues can be recognized: the peripheral tissue and the central tissue. Whereas the central tissue is composed mainly of large infected cells intercalated with smaller, vacuolated uninfected cells, the peripheral tissue includes: from the outside to the inside, the outer cortex, the nodule endodermis, and the inner cortex (also called the nodule parenchyma), which contains the vascular bundles [4].

Several plant proteins involved in this symbiotic process show a specific or enhanced expression pattern in root nodules. These proteins are collectively termed nodulins and have been classified as early or late nodulins according to the timing of their expression during root nodule development [5-7]. In general, early nodulins are involved in initial signaling events, infection development, and nodule organogenesis, whereas late nodulins, which are induced just before or during the onset of the N_2 _fixation process, are involved mainly in nodule metabolism and function.

Large-scale transcriptome analyses conducted in the last decade have enabled the identification of plant peptidases whose expressions are up-regulated during rhizobium infection, nodule development and/or senescence [8-13], suggesting roles for these proteins in the symbiotic process.

Peptidases cleave covalent peptide bonds of proteins or peptides [14], an essential post-translational modification that alters the half-lives, subcellular trafficking and activities of a wide array of proteins [15]. In consequence, peptidases are potentially involved in a multitude of biological processes ranging from simple digestion of proteins to highly-regulated signaling cascades.

Plant aspartic peptidases (APs; EC 3.4.23), a relatively small class of endopeptidases, are composed of either one or two chains [16]. Their catalytic centre is formed by two Asp residues that activate a water molecule, and this event mediates the nucleophilic attack on the peptide bond [14]. Enzymes of this group are active at acidic pH and are generally inhibited by pepstatin A [16]. Although the biological function of most plant APs remains hypothetical, these enzymes have been implicated in protein processing and/or degradation, plant senescence and programmed cell death, stress responses, and reproduction [17].

APs are synthesized as inactive precursors (also known as zymogens), in which a hydrophobic N-terminal signal sequence is followed by a prosegment of about 40 amino acids. Finally, the N- and C-terminal domains are separated by an insertion of 100 amino acids, a plant-specific insert (PSI) present exclusively in most plant APs [17].

A small number of plant APs do not contain a PSI and in consequence have been cataloged as "atypical APs": nucellin and PCS1 (Gi 2290201 and Gi 15241713, respectively) involved in cell death regulation [18,19], CND41 and nepenthesins I and II (Gi 2541876, Gi 41016421 and Gi 41016423, respectively) involved in nitrogen remobilization [20,21], and CDR1 (Gi 37935737), involved in disease resistance [22]. Despite having low sequence identity among them, plant atypical APs contain a high number of cysteines and show specific localizations, which clearly differentiate them from the majority of plant APs [23].

In this study, we report the isolation and characterization of PvNod41, a novel aspartic peptidase from common bean (*Phaseolus vulgaris *L.) that can be classified as a plant atypical AP. PvNod41 shows peptidase activity against casein at mildly acidic pH and is only partially inhibited by pepstatin A. Sequence analysis of PvNod41 revealed that it is closely related to CDR1, an atypical Arabidopsis AP involved in pathogen defense. Considering its biochemical properties, as well as its restricted spatial and temporal expression pattern in uninfected cells of the symbiotic nodule, PvNod41 could play an important role in plant defense during nodule development.

## Results

### Purification of nodulin 41 (PvNod41) and determination of its primary structure

**Figure 1:**
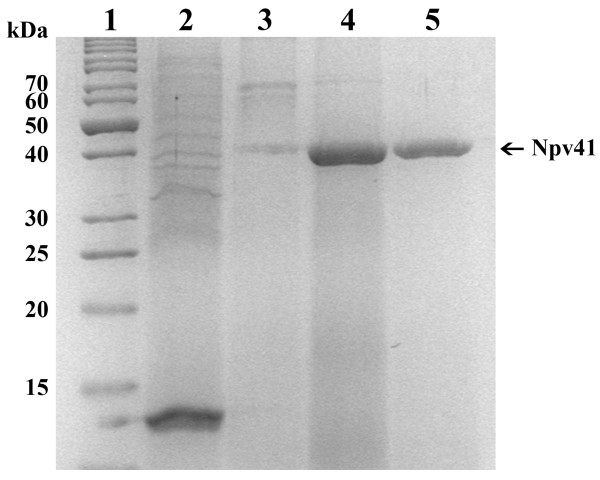
**Analysis of purified PvNod41**. Protein profile on a Coomassie-stained 12% SDS-PAGE gel of collected fractions obtained during PvNod41 purification. Lane 1, protein marker; lane 2, crude protein extract from root nodules; lane 3, 1 M KCl washing; lane 4, fraction A (elution from the BSA-Affi-Gel 10 Gel column); lane 5, fraction B (flow-through of the chromatography on Affi-Gel Heparin Gel).

PvNod41 was first detected in an attempt to isolate root nodule proteins able to interact with a synthetic peptide derived from the amino acid sequence of nodulin 30 [24]. After several interaction assays employing different experimental conditions, we realized that PvNod41 was binding to denatured polypeptides. Accordingly, a method to purify PvNod41 from common bean root nodules was developed, based on a denatured BSA-affinity chromatography column, followed by Affi-Gel Heparin Gel chromatography (Figure 1). 12% SDS-PAGE analysis of the purified protein fraction confirmed the presence of a protein with an apparent molecular mass of 41 kDa. The fraction containing PvNod41 (Figure 1, lane 5), was collected and used for amino acid sequencing, interaction assays and proteolytic activity assays. The calculated purification factor from the crude extract was 250-fold.

**Figure 2:**
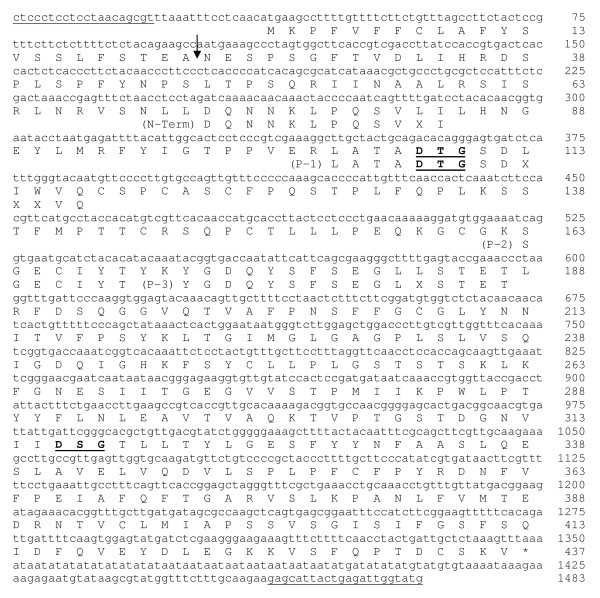
**PvNod41 primary sequence**. PvNod41 gene sequence (lower case) and protein sequence (upper case). *PvNod41 *encodes a 437 amino acid single polypeptide containing Asp-Thr-Gly and Asp-Ser-Gly sequences (DTG and DSG). Conserved motifs around the two catalytic aspartic acid residues are shown in boldface and underlined. Primer sequences used for PCR amplification are underlined. The arrow indicates the cleavage position of the putative signal peptide that directs the protein to the ER. HPLC-purified peptide sequences obtained from the trypsin digestion of PvNod41 [N-terminal end (N-term) as well as three internal peptides (P-1, P-2 and P-3)] are also depicted in this figure. The stop codon is marked with an asterisk.

The identity of PvNod41 was partially determined by Edman degradation from purified trypsin-digested peptides (Figure 2). All of the partial amino acid sequences of PvNod41 were further identified in different expressed sequence tags (ESTs) of common bean (EST database at NCBI, http://blast.ncbi.nlm.nih.gov/Blast.cgi), a fact that allowed us to deduce a virtually complete gene sequence, depicted in detail in Figure 2. Two primers were designed to amplify *PvNod41 *by PCR. A single ~1.5 kb PCR amplification product was obtained using either genomic DNA or cDNA of common bean as template, indicating that this gene contains no introns. The *PvNod41 *gene (GenBank: JN255164.1) encodes a 437 amino acid plant AP (GenBank: AEM05966.1) composed of a single polypeptide chain belonging to the A1B subfamily [16]. The two catalytic sequence motifs in APs (DTG and DSG) are present in the primary sequence of PvNod41 (Figure 2), as is a putative signal peptide of 22 amino acids likely to be responsible for its translocation to the endoplasmic reticulum (ER) [17]. By comparing the deduced amino acid sequence of the PvNod41 genomic clone and the N-terminal amino acid sequence of the PvNod41 purified protein, it became evident that the 50 amino acid prosegment had been removed (Figure 2). Well-known representatives of the A1 peptidase family are generally secreted from cells as inactive zymogens that activate autocatalytically at acidic pH to yield the active peptidase [25]. As we could not find any intermediate form during the purification process (Figure 1), the 50 amino acid N-terminal prosegment of PvNod41 is likely removed by autocatalysis [17].

**Figure 3:**
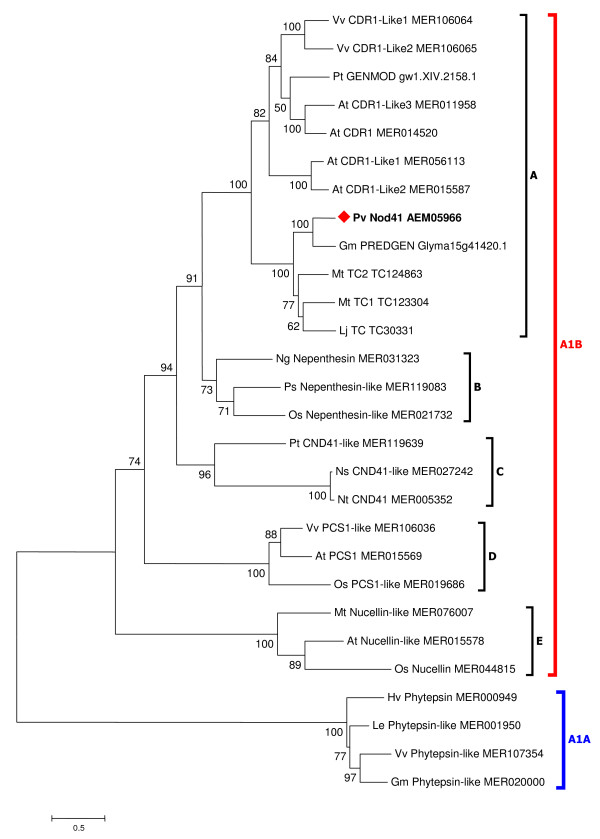
**Relationship of PvNod41 to other plant aspartic proteases**. Phylogenetic relationship between PvNod41 and aspartic peptidases of the A1B subfamily. Groups of representative aspartic peptidases such as CDR1 (A), nepenthesin (B), CND41 (C), PCS1 (D) and nucellin (E), were used for the analysis. Phytepsins of peptidase subfamily A1A were included as an outgroup. Database accession numbers are indicated. The phylogenetic tree was constructed using the Maximum Likelihood method based on protein sequences. Numbers represent number of substitutions per site along the branch. At, *Arabidopsis thaliana*; Gm, *Glycine max*; Hv, *Hordeum vulgare; *Le, *Lycopersicon esculentum*; Lj, *Lotus japonicus; *Mt, *Medicago truncatula; *Ng, *Nepenthes gracilis*; Ns, *Nicotiana sylvestris*; Nt, *Nicotiana tabacum*; Os, *Oryza sativa*; Ps, *Picea psitchensis*; Pt, *Populus trichocarpa*; Pv, *Phaseolus vulgaris*; Vv, *Vitis vinifera*.

A phylogenetic analysis was carried out including selected plant AP sequences representing different groups within the A1B subfamily. Four phytepsins belonging to the A1A subfamily, which are APs with rather different amino acid sequences, were included as an outgroup. Based on this analysis, PvNod41's most closely related protein is CDR1 (43% identity), an AP involved in resistance to pathogens in Arabidopsis and rice [22,26] (Figure 3 and Additional file 1), whereas the other APs were found in different clades (Figure 3).

### Preferential binding of PvNod41 to denatured proteins and peptidase activity

**Figure 4:**
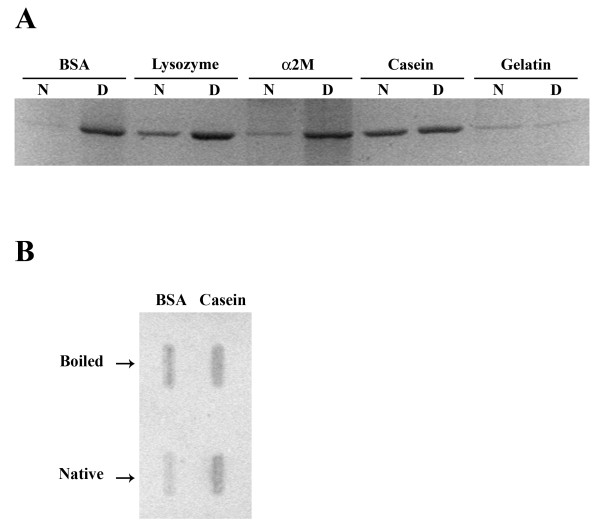
**Preferential binding of PvNod41 to denatured proteins**. (A) PvNod41 binding assay. Purified PvNod41 was incubated with either native (N) or denatured (D) proteins pre-immobilized on agarose-beads. After incubation, samples were extensively washed with PBS. PvNod41 that was bound to immobilized proteins on the matrix was recovered by boiling the sample with Laemmli buffer and analyzed by 12% SDS-PAGE and Coomassie Brilliant Blue staining. BSA, Bovine Serum Albumin; α2M, α2-Macroglobulin. (B) Far western blot assay. Bovine serum albumin (BSA) and casein, either native or denatured by boiling were blotted onto nitrocellulose, probed with purified PvNod41, and immunodetected with anti-PvNod41 antiserum as described in the Methods section.

In order to determine its binding preferences, PvNod41 was incubated with native or denatured model substrates. As shown in Figure 4A, PvNod41 preferentially bound to the denatured forms of BSA, lysozyme and α_2_-macroglobulin, whereas it bound to denatured and native casein to equivalent levels. PvNod41 was unable to bind to an unstructured protein such as gelatin, a mixture of peptides and proteins produced by partial hydrolysis of collagen generally used to evaluate peptidase activity. PvNod41's binding preferences for denatured or native BSA and casein were confirmed in far western blot assays (Figure 4B).

**Figure 5:**
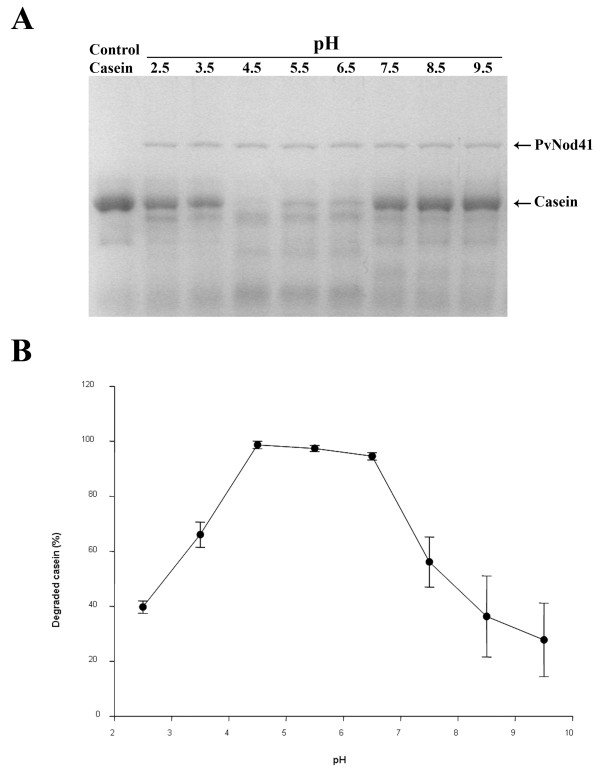
**Effect of pH on the activity of PvNod41**. (A) Purified PvNod41 was tested for activity using casein as a substrate (1 h at 37°C) at pH values ranging from 2.5 to 9.5. Obtained samples were analyzed by 12% SDS-PAGE and stained with Coomassie Blue. (B) Densitometry analysis of degraded casein. Percentage (%) of degraded casein relative to control casein was plotted against pH. Means of three independent experiments ± SE are shown.

Although purified PvNod41 selectively bound to denatured proteins, no peptidase activity was detected on BSA or gelatin at pH 4.5 (Table 1). However, PvNod41 was able to degrade casein in both conformational states (58% of native casein and 67% of acid-denatured casein, compared to the levels degraded by trypsin) (Table 1). The optimal pH of PvNod41 catalytic activity was determined on casein, a classic protease substrate (Figure 5). PvNod41 was found to be most active at pH 4.5 in the assays, although it maintains residual activity at a wider range of pH values (pH 3.5-7.5; Figure 5). Similar data were also obtained by using a chromogenic method that employs succinylated casein as a substrate (QuantiCleave™ Peptidase Assay kit, Pierce). The maximum activity detected by this method was at pH 5.5 (see Additional file 2).

**Table 1:** Semi-quantitative assay of purified PvNod41 proteolytic activity

Protein substrates	Efficiency of cleavage (n = 5)
Casein	58% (± 2%)
Denatured casein	67% (± 5%)
BSA	n.c.
Denatured BSA	n.c.
Gelatin	n.c.

n.c. not cleaved.

Proteolytic activity of purified PvNod41 was tested against several model substrates. The assays were performed as described in "Methods". Efficiency of cleavage was compared to that of trypsin.

The effects of distinct class-specific inhibitors of known peptidases on PvNod41 activity were studied and the results are shown in Table 2. None of the AP inhibitors used could completely abolish the hydrolytic activity of PvNod41 on casein. Inhibition in response to pepstatin A (a widely used inhibitor of APs) was partial, as was that of 2-mercaptoethanol and Fe^3+^. The effect of SDS, known to stimulate peptidase activity, was also deleterious. As expected, EDTA, an inhibitor of metallopeptidase activity, had no effect on PvNod41.

**Table 2:** Proteolytic activity of purified PvNod41

Inhibitor	Concentration	% of residual activity (n = 3)
Pepstatin A	2 μM	55 (± 5)
2-mercaptoethanol	25 mM	62 (± 5)
Fe^3+^	10 mM	39 (± 4)
SDS	0.05%	47 (± 5)
EDTA	5 mM	100

Purified PvNod41 was tested for activity using casein as a substrate in 50 mM sodium citrate, pH 4.5 at 37°C. The enzyme was preincubated in the presence of the indicated inhibitor for 15 min at 37°C before adding the substrate.

### PvNod41 expression pattern in different bean organs and immunolocalization in root nodules

**Figure 6:**
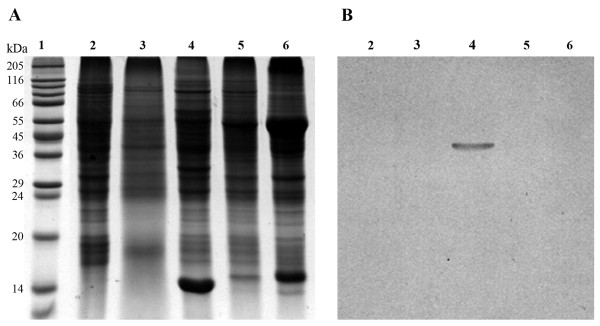
**PvNod41 is expressed exclusively in N2-fixing root nodules of common bean**. (A) 12% SDS-PAGE analysis of crude protein extracts from selected bean tissues. Lane 1, protein marker; lane 2, 3-d-old uninoculated roots; lane 3, 21 days post inoculation (dpi) nodule-stripped roots; lane 4, 21 dpi root nodules; lane 5, stems from 21 dpi plants; lane 6, leaves from 21 dpi plants.(B) Western blot analysis of samples used in A with the anti-PvNod41 antiserum.

**Figure 7:**
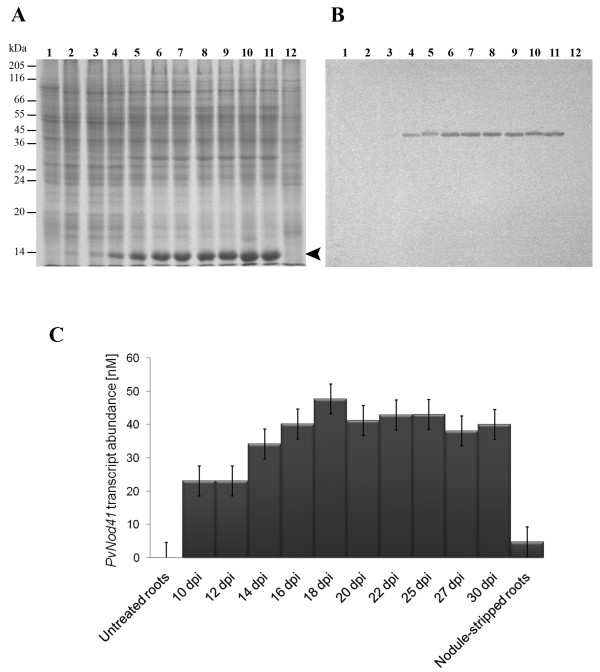
**PvNod41 is a late nodulin**. (A) 12% SDS-PAGE analysis of crude protein extracts from roots and root nodules. Lane 1 and 12, crude protein extracts from 3-d-old uninoculated roots and 21 days post inoculation (dpi) nodule-stripped roots, respectively. Lanes 2 to 11, crude extracts from 10 (lane 2), 12 (lane 3), 14 (lane 4), 16 (lane 5), 18 (lane 6), 20 (lane 7), 22 (lane 8), 25 (lane 9), 27 (lane 10) and 30 (lane 11) dpi root nodules. Arrowhead indicates the accumulation of leghemoglobin during nodule ontogeny. (B) Western blot analysis of the same samples using the anti-PvNod41 antiserum. (C) Accumulation of *PvNod41 *transcripts during nodulation. Equivalent samples to A and B were analyzed by RT-qPCR to determine *PvNod41 *gene expression levels. Eight technical replicates were analyzed per sample. Error bars represent the standard error.

A specific antiserum raised in mouse against purified PvNod41 detected a single 41 kDa band in a crude extract of root nodule proteins, but no signal was detected in similar extracts from roots, nodule-stripped roots, stems, or leaves (Figure 6), confirming that PvNod41 is indeed a nodulin. The temporal expression pattern of PvNod41 during root nodule development was also investigated. No signal was detected in 3-d-old uninoculated roots, 21 days post-inoculation (dpi) nodule-stripped roots, or 10 dpi root nodules (Figure 7). PvNod41 was just barely detected in 12 dpi root nodules, and accumulated in 14 to 30 dpi root nodules (Figure 7). Based on the fact that PvNod41 shows a late developmental expression pattern during root nodule development, correlating with other late nodulins such as leghemoglobin and uricase II [27], this protein should be considered a late nodulin. Additionally, *PvNod41 *transcript accumulation levels were determined by RT-qPCR. *PvNod41 *transcripts were found in 10 to 30 dpi root nodules, whereas no transcripts were detected in 3 d-old uninoculated roots. 21 dpi nodule-stripped roots contained a lower amount of transcript than did root nodules (Figure 7C).

**Figure 8:**
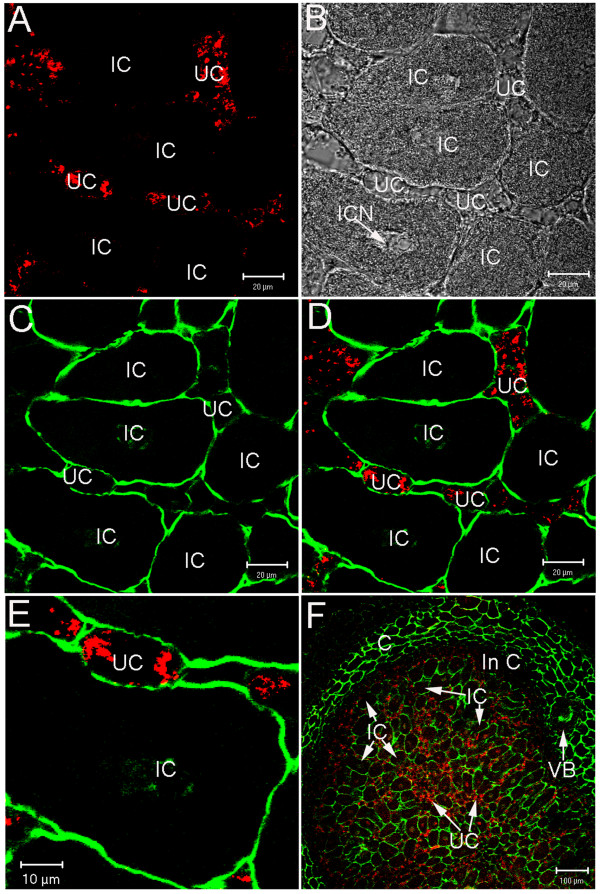
**PvNod41 protein is located in uninfected cells**. Immunolocalization of PvNod41 in root nodule transverse sections with counterstained cell walls. (A) anti-PvNod41 antibodies visualized with a secondary antibody conjugated to Alexa Fluor^® ^633 (red); (B) differential interference contrast (DIC) image; (C) cell wall staining (green); (D) merge of A and C; (E) Image magnification of an uninfected cell of D; (F) Immunolocalization of PvNod41 at whole root nodule level. The images were taken by laser scanning confocal microscopy. IC, Infected Cell; UC, Uninfected Cell; ICN, Infected Cell Nucleus; C, Cortex; In C, Inner Cortex; VB, Vascular Bundle.

Since the bean root nodule is formed by different tissues, each composed of particular cell types, we wanted to know if PvNod41 is expressed in different cells throughout the root nodule or only in a particular cell type. The anti-PvNod41 antiserum was used to specifically detect PvNod41 in root nodule sections by laser scanning confocal microscopy. The PvNod41 signal was restricted to the central tissue of mature nodules (Figure 8F), specifically in uninfected cells (Figure 8 and Additional file 3). PvNod41 signal was not associated with the cell wall, plasma membrane, or vacuole (Figure 8E). Instead, this protease displayed a punctate subcellular distribution that could be indicative of the endomembrane system. Interestingly, the distribution pattern of PvNod41 within the cell (Figure 8E) is similar to that of PCS1, an atypical AP of *Arabidopsis thaliana *that is localized to the ER [19].

## Discussion

Proteolytic enzymes are usually associated with nutrient remobilization during starvation, and senescence, stress responses, and differentiation of cell components [15,28,29]. However, novel findings on plant peptidase functions have revealed their involvement in a broad range of inducible cellular processes [15,30].

A variety of up-regulated genes encoding members of the large peptidase family have been discovered during all stages of the legume-rhizobium symbiosis [8-13], suggesting that peptidases may play an important role in the symbiotic process. Indeed, rhizobium-induced peptidases have been isolated from various nodulating plants. MtMMPL1, a *Medicago truncatula *matrix metalloendoproteinase has been shown to be involved in the *Sinorhizobium meliloti *infection process [31]. *cg12*, a subtilisin-like serine peptidase gene from *Casuarina glauca*, was shown to be specifically expressed during plant cell infections induced by *Sinorhizobium meliloti *in transgenic *Medicago truncatula *plants [32], whereas Sbts, a *Lotus japonicus *serine peptidase of the subtilase superfamily, is transiently expressed during the first two weeks after inoculation with *Mesorhizobium loti *and is proposed to be involved in nodule formation and maintenance [33]. Cysteine peptidases have been implicated in molecular processes such as defense against root invasion by soil microorganisms, protein turnover to create new tissues, cellular homeostasis, and metabolism [34]. In addition, some of them have been identified in the cytoplasm of infected nodule cells and their activity appears to increase markedly during senescence [34,35].

In this work we describe a novel nodulin that has aspartic peptidase (AP) activity and is expressed exclusively in nitrogen-fixing root nodules during the symbiosis of *Phaseolus vulgaris *with rhizobia (Figure 6). Even though AP activity has been previously observed during nodule senescence [36], to our knowledge this is the first time that a specific AP has been isolated and characterized during nodule development.

Partial protein sequencing and *in silico *translation indicated that *PvNod41 *encodes a 437 amino acid single polypeptide containing Asp-Thr-Gly and Asp-Ser-Gly sequences (DTG and DSG, underlined in Figure 2). DTG and DSG are conserved motifs found in all plant APs and are responsible for their catalytic activity. Similarity searches of PvNod41 indicate that this protein indeed belongs to the A1B peptidase subfamily (MEROPS peptidase database, http://merops.sanger.ac.uk/) and shares significant sequence similarity with a plant atypical AP, CDR1, a protein involved in pathogen defense in *Arabidopsis thaliana *(Figure 3 and Additional file 1) [22].

The biochemical characterization of PvNod41 indicates that this enzyme displays unique enzymatic properties, as compared to other APs. Although PvNod41 is able to bind to a variety of denatured peptidase model substrates (Figure 4), it only partially cleaves casein at mildly acidic pH values (Table 1 Figure 5). Similar to CDR1 and also PCS1, another atypical AP involved in cell survival [19], PvNod41 is most active at mildly acidic pH and is incompletely inhibited by the archetypical AP inhibitor pepstatin A (Table 2).

Plant atypical APs are distinguished from typical APs by the absence of the plant-specific insert (PSI). Whereas the PSI is not involved in the catalytic activity of plant APs, it is definitively required for vacuolar localization [37]. Indeed, most typical APs accumulate inside protein storage vacuoles [17]. By contrast, characterized plant atypical APs display unexpected localizations; for example, tobacco CND41 is located in chloroplast nucleoids [38], APs from *Nepenthes *are secreted to the pitchers [21], and Arabidopsis PSC1 is retained in the ER [19]. Likewise, PvNod41 expression is induced in common bean exclusively during root nodule development (Figure 7) and has a specific subcellular localization (Figure 8).

Startlingly, in spite of its sequence similarity to CDR1, PvNod41 is not an extracellular AP. Instead, this particular AP is located exclusively in uninfected cells of the root nodule central tissue (Figure 8), and its pattern of distribution within the cell (Figure 8E) resembles that of Arabidopsis PCS1, which is localized to the ER [19]. Arabidopsis PCS1 and PvNod41 share some other characteristics: both enzymes are able to hydrolyze casein but are inactive against other peptidase model substrates, both are most active at a mildly acidic pH but retain residual activity at a wider range of pH values, and both are only partially inhibited by pepstatin A.

Whereas the biological role of PvNod41 is still unknown, it is tempting to speculate that this protein might contribute to maintaining the integrity of uninfected root nodule cells via a mechanism analogous to that of CDR1 [22]. In the central zone of bean root nodules, interconnected rows of uninfected cells are arranged throughout the central region in such a way that they are in direct contact with virtually all infected cells [4]. In this scenario, the putative peptide produced by the activity of PvNod41 could induce a mild defense response in uninfected cells, which in turn could constrain the spread of the bacteria out of the infected cells of the root nodule. The induction of PvNod41 during nodulation in both effective and ineffective nodules (Figure 7 and data not shown) in addition to its absence from uninfected roots supports the hypothesis that PvNod41 is involved in defense.

Future identification of loss-of-function and gain-of-function mutants, as well as the identification of the natural substrate of PvNod41, will be necessary to understand better the functional role of this enzyme during nodulation.

## Conclusions

Although a large number of plant AP-like proteins have been identified, so far only a few of them have been isolated and characterized. In this work we isolate and characterize a novel nodulin of *Phaseolus vulgaris *with AP activity. PvNod41 is expressed exclusively during the symbiotic process in root nodules and is confined to the uninfected cells of the nodule central zone. Here, we have cloned and purified PvNod41, and our results indicate that this enzyme displays some unique properties and others that are shared by Arabidopsis CDR1 and PCS1, two atypical APs involved in cell defense and survival.

## Methods

### Plant material

Seeds of common bean (*Phaseolus vulgaris *L. cv. Negro Jamapa) were surface sterilized with a solution of 10% (v/v) commercial bleach, rinsed with plenty of water and allowed to germinate for three days on water-saturated towels in the dark at 28°C. Seedlings were then transferred to vermiculite, inoculated with *Rhizobium tropici *CIAT899 [39] and grown in the greenhouse. 3-d-old roots, as well as root nodules, stems, leaves and nodule-stripped roots from 21-days-post-inoculation (dpi) plants were harvested, immediately frozen in liquid nitrogen, and stored at -70°C until use.

### Protein extraction and purification of PvNod41 protein

To prepare crude protein extracts, 5 g of 21 dpi root nodules were frozen in liquid nitrogen, ground with a mortar and pestle to a fine powder, and mixed for 10 min at 4°C in 50 ml of phosphate-buffered saline (PBS) buffer (137 mM NaCl, 2.7 mM KCl, 4.3 mM Na_2_HPO_4_, 1.4 mM KH_2_PO_4_, pH 7.3) containing 2% (w/v) polyvinyl-polypyrrolidone (PVPP). The homogenate was then centrifuged at 12, 000 g for 10 min and the supernatant was recovered.

For PvNod41 purification, bovine serum albumin (BSA) was immobilized on Affi-Gel 10 Gel (Bio-Rad Laboratories, Hercules, CA, USA) according to the manufacturer's instructions and transferred to a column. Coupled BSA was denatured by washing with 5 volumes of 100 mM NaOH. The column was later equilibrated with 20 volumes of PBS buffer. The protein extract was passed through the column and unbound and weakly bound proteins were washed off of the column with 20 volumes of PBS buffer, followed by 5 volumes of 1 M KCl, 10 mM NH_4_OH. PvNod41 was eluted with 100 mM NH_4_OH and 150 mM NaCl. This fraction was immediately neutralized by the addition of Tris-HCl pH 6.8 (250 mM final concentration), and then concentrated by precipitation with 80% ammonium sulfate. After centrifugation (12, 000 g for 10 min at 4°C) the protein pellet was recovered and re-suspended in 1 ml of PBS buffer, de-salted against PBS (generating fraction A, see Figure 1), and passed through an Affi-Gel Heparin Gel column (Bio-Rad Laboratories, Hercules, CA, USA) previously equilibrated with PBS buffer. Heparin is a linear glycosaminoglycan able to bind to a wide range of proteins with some exceptions, including PvNod41, so it was employed to remove contaminating proteins present in fraction A. The Affi-Gel Heparin Gel flow-through fraction contained PvNod41 that was practically pure (fraction B, Figure 1).

### Amino acid sequencing, PCR amplification and cloning of *PvNod41*

100 μg of pure PvNod41 were digested with 5 μg of trypsin (sequencing grade; Roche, Mannheim, Germany) in 50 mM Tris-HCl pH 8.0 and the resulting peptides were purified by reversed-phase HPLC by using a C-18 analytical column (Vydac, Hesperia, CA, USA). Three selected peptides, as well as the N-terminal end of the entire protein, were sequenced in an automated gas-phase sequencer (LF 3000 Protein Sequencer; Beckman, Fullerton, CA, USA). All partial amino acid sequences were BLASTed against the common bean Expressed Sequence Tag (EST) database (NCBI, http://blast.ncbi.nlm.nih.gov/Blast.cgi;) [40], and a virtually complete gene sequence was generated. Two specific primers aimed at amplifying *PvNod41 *by PCR were designed: 5'- CTCCCTCCTCCTAACAGCGT-3' and 5'-CATACCAATCTCAGTAATGCTC-3'. The amplified PCR product was cloned into the pCR^®^T7/CT-TOPO^® ^expression vector (Invitrogen, Carlsbad, CA, USA) and sequenced by Taq FS Dye Terminator Cycle Sequencing Fuorescence-Based Sequencing in a Perkin Elmer/Applied Biosystems 3730 apparatus to confirm the nucleotide sequence of *PvNod41*.

### Sequence alignment and Phylogenetic analysis

The deduced amino acid sequence of *PvNod41 *was BLASTed against different databases at NCBI, as well as in the MEROPS database, the Glyma1 assembly of the Soybean (*Glycine max*) genome project http://www.phytozome.net/soybean.php, the *Lotus japonicus *and *Medicago truncatula *databases of The Gene Index Project http://compbio.dfci.harvard.edu/tgi/, and the *Populus trichocarpa *database of The Joint Genome Institute http://genome.jgi-psf.org/. Related protein sequences were aligned (ClustalW Multiple Sequence Alignment Program http://www.ch.embnet.org/software/ClustalW.html) and displayed using BOXSHADE 3.21 http://www.ch.embnet.org/software/BOX_form.html.

The eleven protein sequences with the highest identity to PvNod41, as well as representative aspartic peptidases of the A1B subfamily (MEROPS database) were aligned using ClustalX [41]. Four phytepsins members of the A1A subfamily were also included as an outgoup. A phylogenetic tree was constructed using the Maximum Likelihood method based on protein sequences. The topology was inferred using the PHYML package with the WAG substitution matrix (loglk = -22012.58462 1). The tree was edited with MEGA 3.1 software [42].

### Protein binding assays

BSA, lysozyme and α_2_-macroglobulin were immobilized on agarose beads (Affi-Gel 10 Gel, Bio-Rad Laboratories, Hercules, CA, USA) according to the manufacturer's instructions. α-casein-agarose and gelatin-agarose were purchased from Sigma (Sigma-Aldrich, St. Louis, MO, USA). One half of each preparation was treated for 10 min with 100 mM NaOH to induce the denaturation of the bound protein, whereas the second half was untreated, maintaining the protein in its native state. Both samples of each preparation were then abundantly washed using 20 volumes of PBS buffer. 50 μl of each sample (with native or denatured proteins) were incubated for 1 h at room temperature with purified PvNod41. After extensive washing with PBS buffer, PvNod41 that was bound to immobilized proteins on the matrix was recovered by boiling the sample with Laemmli buffer 2× [125 mM Tris-HCl pH 6.8, 4% SDS, 20% glycerol, 10% 2-mercaptoethanol, 0.02% (w/v) bromophenol blue] and analyzed by 12% SDS-PAGE and Coomassie Blue staining.

PvNod41 binding preferences to BSA and casein were also evaluated using a far western blot assay (also known as an overlay assay). Briefly, 20 μg of native or denatured casein or BSA were transferred by vacuum to nitrocellulose membranes (Hybond-C Extra; Amersham Biosciences, Little Chalfont, Bucks, UK). Blotted membranes were blocked for 1 h at room temperature in 0.5% Triton X-100 dissolved in Tris-Buffered Saline (TBS) buffer (30 mM Tris pH 8.0, 150 mM NaCl) and incubated for 3 h at room temperature with 10 μg/ml of PvNod41 dissolved in TBST [TBS, 0.1% (v/v) Triton X-100]. After washing three times for 15 min each with TBST, the blots were incubated with anti-PvNod41 antiserum diluted 1:5000 in TBST. Immunodetection of PvNod41 was performed according to the western blot protocol described below.

### Peptidase activity assay

Proteolytic activity of PvNod41 was tested against several model substrates such as casein, BSA, and gelatin according to established protocols. Briefly, 10 μg of native or trichloroacetic acid (TCA)-denatured casein, or native or TCA-denatured BSA, were mixed with 300 ng of PvNod41 and incubated for 1 h at 37°C in 50 mM of sodium citrate, pH 4.5. The same assay was carried out using 300 ng of TPCK Trypsin (EC 3.4.21.4) (QuantiCleave™ Peptidase Assay kit; Pierce, Rockford, IL, USA) as a positive control in 50 mM sodium borate, pH 8.5. Proteolysis was evaluated by densitometry analysis of Coomassie blue-stained bands after 12% SDS-PAGE.

The activity towards gelatin was assayed in-gel as follows: 200 ng of PvNod41 were mixed with Laemmli buffer 2× and loaded, without reducing or boiling, on a 10% SDS-Polyacrylamide gel copolymerized with 0.15% gelatin. The gel was run at constant voltage (120 V) for 1.5 h at room temperature. SDS was removed by washing the gel three times with 50 ml of 20 mM Tris-HCl, pH 4.5 with 0.1% (v/v) Triton X-100 for 30 min at room temperature and incubation overnight at 37°C. Finally, the gel was stained with Coomassie blue stain for 1 h, followed by de-staining. Proteolytic activity appeared as clear bands on a blue background.

For activity assays at different pH values, 10 μg of casein was mixed with 300 ng of PvNod41 and incubated for 1 h at 37°C in 50 mM of the appropriate buffer (glycine-HCl, pH 2.5; sodium citrate, pH 3.5-5.5; potassium phosphate, pH 6.5 or Tris-HCl, pH 7.5-9.5). Proteolysis was measured as described above.

Alternatively, PvNod41 peptidase activity was evaluated using the QuantiCleave™ Peptidase Assay kit (Pierce, Rockford, IL, USA) following the manufacturer's instructions. Activity assays were also performed at different pH values. 200 ng of PvNod41 were incubated overnight at room temperature with succinylated casein in the presence of 50 mM of the appropriate buffer (sodium citrate, pH 4.5-5.5; sodium phosphate, pH 6.5-7.5; or sodium borate, pH 8.5-9.0). After digestion, the fragments were separated by 12% SDS-PAGE, stained with Coomasie blue and analyzed by densitometry as described.

The effect of diverse peptidase inhibitors such as EDTA and pepstatin A, among others, was tested by preincubating PvNod41 with the inhibitor for 15 min at 37°C before adding casein. Samples were incubated for 1 h at 37°C. The amount of residual casein was determined by 12% SDS-PAGE.

### Raising of PvNod41 antiserum and western blotting

13 μg of pure PvNod41 were mixed with 100 μl of complete Freund's adjuvant (Gibco-BRL, Grand Island, NY, USA) and injected subcutaneously to BALB/c mice. Boost injections were prepared in the same manner but using incomplete Freund's adjuvant instead, and were administered at two-week intervals. The antiserum was obtained 14 days after the last injection.

Plant tissue samples were resolved by 12% SDS-PAGE and electrophoretically transferred to nitrocellulose membranes (Hybond-C Extra; Amersham Biosciences, Little Chalfont, Bucks, UK). Blotted membranes were blocked for 1 h at room temperature in 5% (w/v) nonfat dried milk dissolved in Tris-Buffered Saline Triton-X100 (TBST) buffer [30 mM Tris pH 8.0, 150 mM NaCl, 0.1% (v/v) Triton X-100] and incubated for 1 h at room temperature with anti-PvNod41 antiserum diluted 1:5000 in TBST. After washing three times for 15 min each with TBST, the blots were incubated for 1 h at room temperature with goat anti-mouse IgGAM (H+L) coupled to alkaline phosphatase (Zymed Laboratories, Inc., San Francisco, CA, USA) diluted 1:5000 in TBST, washed again three times for 15 min each with TBST, and developed with NBT and BCIP (Zymed Laboratories, Inc., San Francisco, CA, USA).

### RNA isolation and quantitative RT-PCR

Total RNA was isolated using Trizol reagent (Invitrogen, Carlsbad, CA, USA). RNA samples were treated with DNase I (Invitrogen) followed by cDNA synthesis using the Revert Aid H Minus first strand cDNA synthesis kit (Fermentas, St. Leon-Rot, Germany). Gene-specific primers to generate 140-150 bp PCR products were designed using the OligoPerfect™ (Invitrogen) software (Table 3). Real-time RT-PCR reactions were performed in optical reaction tubes using an iCycler iQ5 apparatus (BioRad, Hercules, CA, USA). *PvNod41 *transcript levels were determined with Maxima™ SYBR Green qPCR Master Mix (Fermentas) according to the manufacturer's protocol in a final volume of 15 μl. The cycling conditions were: preheating for 5 min at 95°C followed by 40 cycles (denaturing for 15 s at 95°C, annealing and elongation for 15 s at 55.8°C and data acquisition at 81°C). A negative control reaction without template was also included for each primer combination. The melting curve protocol began immediately after amplification and consisted of 1 min at 55°C followed by 80-10 s steps with a 0.5°C increase in temperature at each step. Threshold values for threshold cycle (Ct) determination were generated automatically by the iCycler iQ5 software. Eight technical replicates were analyzed for each biological replicate. Transcript amounts of *PvNod41 *in each sample were obtained by comparison to a *PvNod41 *standard curve. The standard curve was prepared by serial dilutions of a known plasmid concentration containing the coding sequence of *PvNod41*. Primer and cycling conditions were performed as above described. Each standard point had six technical replicates. Additionally, a similar analysis was performed using the bean elongation factor (*PvEf1*-α) as a reference gene due to its minimal variability between different treatments. Similar *PvNod41*expression data were obtained (data not shown).

**Table 3:** Primer sequences used for RT-qPCR

Primer name	Accession number	Primer sequence (5'→3', forward, reverse)
Pv_Ef1-alpha Fwd	CV530481	GGTCATTGGTCATGTCGACTCTGG
Pv_Ef1-alpha Rv		GCACCCAGGCATACTTGAATGACC
Pv_Nod41 Fwd	JN255164	TTCACAAATCGGTGACCAAATCG
Pv_Nod41 Rv		AACCACGGTTTCATTATCATCGG

### Immunohistochemistry

Freshly harvested 20 dpi *P. vulgaris *root nodules were collected, fixed, dehydrated, methacrylate-embedded, and polymerized as previously reported [43]. For the visualization of cell walls, a method based on the modified pseudo-Schiff propidium iodide (mPS-PI) staining technique [44], with some further modifications, was followed. Re-hydrated nodule sections were incubated in 1% periodic acid at 40°C for 20 min and washed five times for 5 min each with distilled water. The sections were then incubated in Schiff reagent (100 mM sodium metabisulphite, 0.15 N HCl) for 40 min at room temperature and washed twice with distilled water. Following, 50 μg/ml propidium iodide incubation for 10 min at room temperature was performed. Then, sections were washed three times with distilled water. The PvNod41 immunolocalization was performed on these sections. Nodule sections were blocked for 2 h at room temperature in 5% (w/v) nonfat dried milk dissolved in Tris-Buffered Saline Tween 20 (TBST) buffer [0.01 M Tris pH 8.0, 0.15 M NaCl, 0.05% (v/v) Tween 20] and incubated overnight at 4°C with the anti-PvNod41 antiserum diluted 1:50 (v:v) in TBST plus 5% (w/v) nonfat dried milk. After washing three times for 10 min each with TBST, nodule sections were incubated at 4°C for 4 hrs with Alexa Fluor^® ^633 goat anti-mouse IgG H+L (Invitrogen) diluted 1:100 (v/v) in TBST. The sections were washed three times for 10 min each with TBST at 4°C and mounted with Citifluor (Ted Pella, Inc., Redding, CA, U.S.A.). Analysis was performed using a Zeiss LSM 510 Meta confocal microscope (Carl Zeiss Advanced Imaging Microscopy, Jena, Thübingen, Germany) attached to an Axiovert 200 M. Alexa Fluor^® ^excitation was obtained at 633 nm, using a He/Ne laser, a HFT UV 488/543/633 nm dual dichroic excitation mirror and a NFT 545 secondary dichroic beam splitter with a BP 650-710 IR emission filter for detection. Cell walls were observed simultaneously via excitation at 488 nm with an Ar2 laser, using a HFT UV 488/543/633 nm dual dichroic excitation mirror with a LP 505 emission filter. Images were processed using Adobe Photoshop 7.0 software (Adobe Systems Inc., Mountain View, CA, U.S.A.). The images shown were acquired from a single optical section.

In addition, the presence of bacteroids in the infected root nodule cells was confirmed by the Sytox Green nucleic acid staining method [45]. PvNod41 was immunodetected as previously described with a specific antiserum. Anti-PvNod41 antibodies were visualized with a secondary antibody conjugated to Alexa Fluor^® ^633 (red) (Additional file 3). Bacteroid's DNA and nuclei were stained with Sytox Green (green). Uninfected cells, containing PvNod41antigen in the cytoplasm were clearly distinguished from infected cells containing bacteroids, heavily stained with Sytox Green. Nodule sections were obtained from fixed, dehydrated, methacrylate-embedded, and polymerized *Phaseolus vulgaris *root nodules [43].

## Authors' contributions

JEO purified PvNod41, determined its biochemical performance, carried out the immunoassays, and drafted the manuscript. CDC performed the quantitative RT-PCR analysis, designed and wrote the manuscript. GEN isolated the *PvNod41 *gene. XAA carried out the immunohistochemistry, cell walls staining and confocal microscopy analysis. MRK carried out RT-PCR preliminary experiments. FZZ and TOM purified and sequenced the PvNod41 peptides. YM and LS participated in the sequence alignment and worked on the phylogenetic analysis. FS conceived of the study, and participated in designing and coordinating research activities and in drafting the manuscript. All authors reviewed and approved the final manuscript.

## Supplementary Material

Additional file 1 **General structure of PvNod41 (top) and alignment analysis (bottom) with the eleven most similar plant protein sequences found in different databases**. Accession numbers are indicated in parentheses. Pv PvNod41, *Phaseolus vulgaris *Nodulin 41 (AEM05966); Gm PREDGEN, *Glycine max *predicted gene (Glyma15g41420.1); Lj TC, *Lotus japonicus *Tentative Consensus (TC) sequence (TC30331); Mt TC1, *Medicago truncatula *TC sequence 1 (TC123304); Mt TC2, *Medicago truncatula *TC sequence 2 (TC124863); At CDR1-like 1, *Arabidopsis thaliana *CDR1-like sequence 1 (MER056113); At CDR1-like 2, *Arabidopsis thaliana *CDR1-like sequence 2 (MER015587); At CDR1-like 3, *Arabidopsis thaliana *CDR1-like sequence 3 (MER011958); At CDR1, *Arabidopsis thaliana *CDR1 (MER014520); Pt GENMOD, *Populus trichocarpa *gene model (gw1.XIV.2158.1); Vv CDR1-like 1, *Vitis vinifera *CDR1-like sequence 1 (MER106064); Vv CDR1-like 2, *Vitis vinifera *CDR1-like sequence 2 (MER106065). The alignment was done with ClustalW Multiple Sequence Alignment Program http://www.ch.embnet.org/software/ClustalW.html and displayed using BOXSHADE 3.21 http://www.ch.embnet.org/software/BOX_form.html. Gaps were inserted to maximize the similarities. Identical conserved amino acid residues are highlighted in black. Catalytic sequence motifs for aspartic proteases are marked by asterisks and red boxes, whereas cysteines are highlighted in yellow boxes. Click here for file

Additional file 2 **PvNod41 peptidase activity detected with a chromogenic method**. (**A**) Activity of PvNod41 on succinylated casein was assayed by using the QuantiCleaveTM Peptidase Assay kit (Pierce). Purified PvNod41 was incubated overnight at 37°C in different buffers at pH values between pH 4.5 and 9.0. The color produced by peptidase activity was measured at 450 nm and plotted against pH. Results of two independent experiments are shown. (**B**) Representative output of this assay on 12% SDS-PAGE analysis. The gel was stained with Coomassie Blue. Click here for file

Additional file 3 **Immunolocalization of PvNod41 in uninfected cells of common bean root nodule sections**. PvNod41 was immunodetected with a specific antiserum. Anti-PvNod41 antibodies were visualized with a secondary antibody conjugated to Alexa Fluor^® ^633 (red), whereas bacteroids and nuclei were stained with Sytox Green (green). Uninfected cells (UC) containing PvNod41antigen can be clearly distinguished from infected cells (IC) containing bacteroids. ICN, Infected Cell Nucleus; UCN, uninfected cell nucleus. Click here for file
