# Supplementary material for: Nodulin 41, a novel late nodulin of common bean with peptidase activity
Source: BMC Plant Biol. 2011 Oct 10;11:134. doi: 10.1186/1471-2229-11-134 (PMC3207901; doi:10.1186/1471-2229-11-134)
Supplement: Additional file 1 — General structure of PvNod41 (top) and alignment analysis (bottom) with the eleven most similar plant protein sequences found in different databases. Accession numbers are indicated in parentheses. Pv PvNod41, Phaseolus vulgaris Nodulin 41 (AEM05966); Gm PREDGEN, Glycine max predicted gene (Glyma15g41420.1); Lj TC, Lotus japonicus Tentative Consensus (TC) sequence (TC30331); Mt TC1, Medicago truncatula TC sequence 1 (TC123304); Mt TC2, Medicago truncatula TC sequence 2 (TC124863); At CDR1-like 1, Arabidopsis thaliana CDR1-like sequence 1 (MER056113); At CDR1-like 2, Arabidopsis thaliana CDR1-like sequence 2 (MER015587); At CDR1-like 3, Arabidopsis thaliana CDR1-like sequence 3 (MER011958); At CDR1, Arabidopsis thaliana CDR1 (MER014520); Pt GENMOD, Populus trichocarpa gene model (gw1.XIV.2158.1); Vv CDR1-like 1, Vitis vinifera CDR1-like sequence 1 (MER106064); Vv CDR1-like 2, Vitis vinifera CDR1-like sequence 2 (MER106065). The alignment was done with ClustalW Multiple Sequence Alignment Program http://www.ch.embnet.org/software/ClustalW.html and displayed using BOXSHADE 3.21 http://www.ch.embnet.org/software/BOX_form.html. Gaps were inserted to maximize the similarities. Identical conserved amino acid residues are highlighted in black. Catalytic sequence motifs for aspartic proteases are marked by asterisks and red boxes, whereas cysteines are highlighted in yellow boxes. [file 1471-2229-11-134-S1.PDF]

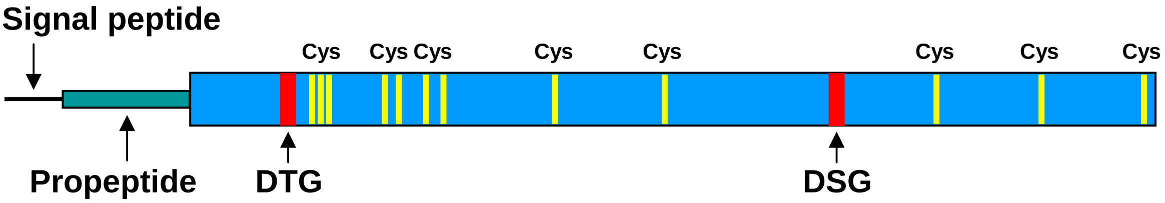


```
Pv Npv41         1 ----MKPFVFFCLAFYS-VSSLFSTEANESP-SGFTVDLIHRDSPLSPFYNPSLTPSQRI
Gm PREDGEN        1 ----MHPWVFMILALFS-LSTLSSREAREGL-RGFSVDLIHRDSPSSPFYNPSLTPSERI
Lj TC             1 ----MHALAFFFAASCSLLATLPFTEPSKTP-SSFTIDLIHHDSPLSPFYNSSMTRSQLI
Mt TC1            1 ----MHAFIFLFSALCS-LYTPSFVESTKNP-SGFEVELIHHDSPLSPFYNSSLTSSELI
Mt TC2            1 ----MDALVFFILALFSLSSTLFIIEASKIQ-SGFSVDLIHRDSPMSPFYNPSMSKLDLI
At CDR1-like 1    1 -----MAT-QILLCFFLFFSVTLSS--SGHPK-NFSVELIHRDSPLSPIYNPQITVTDRL
At CDR1-like 2    1 -----MATKTFLYCSLLAISFFFASNSSANRE-NLTVELIHRDSPHSPLYNPHHTVSDRL
At CDR1-like 3    1 ----MASLIFATLLSLLLLS-----NVNAYPKDGFTIDLIHRDSPKSPFYNSAETSSQRM
At CDR1           1 ----MASLFSSVLLSLCLLSSLFLSNANAKPKLGFTADLIHRDSPKSPFYNPMETSSQRL
Pt GENMOD         1 -----------------------------ARKVGFTVDLIHRDSPLSPFYNSEETDLQRI
Vv CDR1-like 1    1 MEGFSLKFLFYTLAVIFFIHFSGLSHTEASNKGGFSTDLISRDSPLSPFYNPSETQFDRL
Vv CDR1-like 2    1 MEGFNLKFVFCLLAIIFLIYFAKHSQAEAKVDG-FTTDFISRDSPRSPFYNPSETKYQRL

Pv Npv41        55 INAALRSISRLNRVSNLLD-----QNNKLPQSVLILHNGEYLMRFYIGTPPVERLATADT
Gm PREDGEN       55 INAALRSMSRLQRVSHFLD-----EN-KLPESLLIPDKGEYLMRFYIGSPPVERLAMVDT
Lj TC            56 RNAAMRSISRANQLSLSLSHSLNQLKESSPEPIIIPNNGNYLMRIYIGTPSVERLAIADT
Mt TC1           55 TNAALRSISRSKRLSLFQ---NNELNES-PESIIIPNGGDYLMKIYIGTPPVERLAVADT
Mt TC2           56 RNAAFRSKTRLTRFSHSLSLLSNENFQS-TESIIKPNNGDYLMRIYIGTPPVEKLAIFDT
At CDR1-like 1   52 NAAFLRSVSRSRRFNHQ-------LSQTDLQSGLIGADGEFFMSITIGTPPIKVFAIADT
At CDR1-like 2   55 NAAFLRSISRSRRF----------TTKTDLQSGLISNGGEYFMSISIGTPPSKVFAIADT
At CDR1-like 3   52 RNAIRRSARSTLQFSNDDA------SPNSPQSFITSNRGEYLMNISIGTPPVPILAIADT
At CDR1          57 RNAIHRSVNRVFHFTEKDN------TPQ-PQIDLTSNSGEYLMNVSIGTPPFPIMAIADT
Pt GENMOD        32 NNALRRSISRVHHFDPIAAA---SVSPKAAESDVTSNRGEYLMSLSLGTPPFKIMGIADT
Vv CDR1-like 1   61 QKAFHRSISRANHFR---AN---GVSTNSIQSPVISNNGEYLMNISLGTPPVSMHGIADT
Vv CDR1-like 2   60 QKAFRRSILRGNHFR---AI---RASPNDIQSNVISGGGSYLMNISLGTPPVSMLGIADT
                                                                             **

Pv Npv41       110 GSDLIWVQCSPCAS--CFPQSTPLFQPLKSSTFMPTTCRSQPCTLLLPEQKGCGKSG-EC
Gm PREDGEN     109 GSSLIWLQCSPCHN--CFPQETPLFEPLKSSTYKYATCDSQPCTLLQPSQRDCGKLG-QC
Lj TC          116 GSDLTWVQCSPCDNTKCFAQNTPLYDPLNSSTFTLLPCDSQPCTQLPYSQYVCSDYG-DC
Mt TC1         111 GSNLIWVQCSPCK--KCFPQDKPYFDPNKSSTYMGLSCDSQSCSSLPLGKHRCGKSK-KC
Mt TC2         115 GSDLTWVQCSPCIN--CFAQDTPLYDRTKSSSHTNLTCDTQSCTLLPKKQQFCGKSQ-EC
At CDR1-like 1 105 GSDLTWVQCKPCQQ--CYKENGPIFDKKKSSTYKSEPCDSRNCQALSSTERGCDESNNIC
At CDR1-like 2 105 GSDLTWVQCKPCQQ--CYKQNSPLFDKKKSSTYKTESCDSKTCQALSEHEEGCDESKDIC
At CDR1-like 3 106 GSDLIWTQCNPCED--CYQQTSPLFDPKESSTYRKVSCSSSQCRALE-DAS-CSTDENTC
At CDR1        110 GSDLLWTQCAPCDD--CYTQVDPLFDPKTSSTYKDVSCSSSQCTALENQAS-CSTNDNTC
Pt GENMOD       89 GSDLIWTQCKPCER--CYKQVDPLFDPKSSKTYRDFSCDARQCSLLD-QST-CSG--NIC
Vv CDR1-like 1 115 GSDLLWRQCKPCDS--CYEQIEPIFDPAKSKTYQILSCEGKSCSNLGGQGG-CS-DDNTC
Vv CDR1-like 2 114 GSDLIWRQCLPCDD--CYKQVEPLFDPKKSKTYKTLGCNNDFCQDLGQQGS-CG-DDNTC
                   *

Pv Npv41       167 IYTYKYGDQYSFSEGLLSTETLRFDSQGG----VQTVAFPNSFFGCGLYNNITVFPSYKL
Gm PREDGEN     166 IYGIMYGDK-SFSVGILGTETLSFGSTGG----AQTVSFPNTIFGCGVDNNFTIYTSNKV
Lj TC          175 IYAYTYGDN-SYSYGGLSSDSIRFDATAATP------TIPKFVFGCGFQNKFTADKSGKT
Mt TC1         168 EYLIIYGDE-SYSFGELSTDSIGFGSMNGEGEK--DVTFPKSVFGCGLQNDLGSETSHKT
Mt TC2         172 LYSYHYGDK-SFSVGELVVDSISFGSNSGVDVNNVDMTFPKSIFGCGYYNIFTADNSGKT
At CDR1-like 1 163 KYRYSYGDQ-SFSKGDVATETVSIDSASGSP-----VSFPGTVFGCGYNNGGTFDE--TG
At CDR1-like 2 163 KYRYSYGDN-SFTKGDVATETISIDSSSGSS-----VSFPGTVFGCGYNNGGTFEE--TG
At CDR1-like 3 162 SYTITYGDN-SYTKGDVAVDTVTMGSSGRRP-----VSLRNMIIGCGHENTGTFDP--AG
At CDR1        167 SYSLSYGDN-SYTKGNIAVDTLTLGSSDTRP-----MQLKNIIIGCGHNNAGTFNK--KG
Pt GENMOD      143 QYQYSYGDR-SYTMGNVASDTITLDSTTGSP-----VSFPKTVIGCGHENDGTFSD--KG
Vv CDR1-like 1 171 IYSYSYGDG-SHTSGDLAVDTLTIGSTTGRP-----VSVPKVVFGCGHNNGGTFEL--HG
Vv CDR1-like 2 170 TSSYSYGDQ-SYTRRDLSSETFTIGSTEGDP-----ASFPGLAFGCGHSNGGTFNE--KD
```

```
Pv Npv41          223 TGIMGLGAGPLSLVSQIGDQIGHKFSYCLLPLGSTS--TSKLKFGNESIITG----EGVV
Gm PREDGEN        221 MGIAGLGAGPLSLVSQLGAQIGHKFSYCLLPYDSTS--TSKLKFGSEAIITT----NGVV
Lj TC             228 TGIVGLGAGPLSLVSQLGDEIGHKFSYCLLPFSSNS--NSKLKFGEAAIVQG----NGVV
Mt TC1            225 TGIVGLGLGPLSLVSQLGDSIGRKFSYCLVPFGSNS--TSKLKFGDQAIIKG----NGVV
Mt TC2            231 SGLVGLGAGPLSLVSQLGHSIGRKFSYCLVPFGSNS--TSKLTFGNQSTITG----NEVV
At CDR1-like 1    215 SGIIGLGGGHLSLISQLGSSISKKFSYCLSHKSATTNGTSVINLGTNSIPSSLSKDSGVV
At CDR1-like 2    215 SGIIGLGGGPLSLVSQLGSSIGKKFSYCLSHTAATTNGTSVINLGTNSIPSNPSKDSATL
At CDR1-like 3    214 SGIIGLGGGSTSLVSQLRKSINGKFSYCLVPFTSETGLTSKINFGTNGIVSG----DGVV
At CDR1           219 SGIVGLGGGPVSLIKQLGDSIDGKFSYCLVPLTSKKDQTSKINFGTNAIVSG----SGVV
Pt GENMOD         195 SGIVGLGAGPLSLISQMGSSVGGKFSYCLVPLSSRAGNSSKLNFGSNAVVSG----PGVQ
Vv CDR1-like 1    223 SGLVGLGGGPLSMISQLRPLIGGRFSYCLVPVSS------KMHFGSRGIVSG----AGAV
Vv CDR1-like 2    222 SGLIGLGGGPLSLVMQLSSKVGGQFSYCLVPLSSDSTASSKINFGKSAVVSG----SGTV

Pv Npv41          277 STPMIIKPWLPTYYFLNLEAVTVAQKTVPTGS-------------TDGNVIIDSGTLLTY
Gm PREDGEN        275 STPLIIKPSLPTYYFLNLEAVTIGQKVVSTGQ-------------TDGNIVIDSGTPLTY
Lj TC             282 STPLIIKP-DLPFYYLNLEGITVGAKTVKTGQ-------------TDGNIIIDSGSTLTY
Mt TC1            279 STPLMIKSSDPYHYYLNLEGITVGQKTAQSGQ-------------TDGNIIIDSGTTLTY
Mt TC2            285 STPLIIKSLEPTFYFVNLEGITIGQKTIQTGQ-------------IDGNIIIDSGTTLTY
At CDR1-like 1    275 STPLVDK-EPLTYYYLTLEAISVGKKKIPYTGSSYNPNDDGILSETSGNIIIDSGTTLTL
At CDR1-like 2    275 TTPLIQK-DPETYYFLTLEAVTVGKTKLPYTGGGYGLN--GKSSKRTGNIIIDSGTTLTL
At CDR1-like 3    270 STSMVKKD-PATYYFLNLEAISVGSKKIQFTSTIFGTG------E--GNIVIDSGTTLTL
At CDR1           275 STPLIAKASQETFYYLTLKSISVGSKQIQYSGSDSESS------E--GNIIIDSGTTLTL
Pt GENMOD         251 STPLLSSETMSSFYFLTLEAMSVGNERIKFGDSSLGTG------E--GNIIIDSGTTLTI
Vv CDR1-like 1    273 STPLAS-RQPDTFYYLTLESMSVGSKKLAYKGFSKVGSPLADADE--GNIIIDSGTTLTL
Vv CDR1-like 2    278 STPLIK-GTPDTFYYLTLEGMSLGSEKVAFKGFSKNKSSPAAAEE--SNIIIDSGTTLTL
                                                                         ***

Pv Npv41          324 LGESFYYNFAASLQESLA-VELVQDVLSPLPFCFPYRDN-FVFPEIAFQFTGARVSLKPA
Gm PREDGEN        322 LENTFYNNFVASLQETLG-VKLLQDLPSPLKTCFPNRAN-LAIPDIAFQFTGASVALRPK
Lj TC             328 LEESFYNEFVSLVKETVA-VEEDQYIPYPFDFCFTYKEGMSTPPDVVFHFTGGDVVLKPM
Mt TC1            326 LEPKFYNDFIASVKGVIG-VEEVKDPPSPFTFCFTFGDLAKFP-NFVFHFTGADVTLKPQ
Mt TC2            332 LEQPFFDDFVTSVKEAIG-LEEAVDIPSPFHYCFE--DPNSMFPSIVLHFTGANVPLQPK
At CDR1-like 1    334 LEAGFFDKFSSAVEESVTGAKRVSDPQGLLSHCFKSGSAEIGLPEITVHFTGADVRLSPI
At CDR1-like 2    332 LDSGFYDDFGTAVEESVTGAKRVSDPQGLLTHCFKSGDKEIGLPAITMHFTNADVKLSPI
At CDR1-like 3    321 LPSNFYYELESVVASTIK-AERVQDPDGILSLCY-RDSSSFKVPDITVHFKGGDVKLGNL
At CDR1           327 LPTEFYSELEDAVASSID-AEKKQDPQSGLSLCY-SATGDLKVPVITMHFDGADVKLDSS
Pt GENMOD         303 VPDDFFSNLSTAVGNQVE-GRRAEDPSGFLSVCY-SATSDLKVPAITAHFTGADVKLKPI
Vv CDR1-like 1    330 LPQDFYGTLESNVVSAIG-GKPVRDPNNVFSLCY-SNLSGLRIPTITAHFVGADLELKPL
Vv CDR1-like 2    335 LPRDFYTDMESALTKVIG-GQTTTDPRGTFSLCY-SGVKKLEIPTITAHFIGADVQLPPL

Pv Npv41          382 NLFVMTEDRNTVCLMIAPSSVSGISIFGSFSQIDFQVEYDLEGKKVSFQPTDCSKV-
Gm PREDGEN        380 NVLIPLTDSNILCLAVVPSSGIGISLFGSIAQYDFQVEYDLEGKKVSFAPTDCAKV-
Lj TC             387 NTLVLIED-NLICSTVVPSHFDGIAIFGNLGQIDFHVGYDIQGGKVSFAPTDCSLN-
Mt TC1            384 KLLGVLGN-NSYCLLAIPS-ND-LSIFGNIAHVDFLVEYDLEGKKVSFAPSDCSKN-
Mt TC2            389 NVLLLEEN-NLVCLAIVPSNIAGISILGNLAQIDFQVEYDLDGKKLSFAPSDCTKN-
At CDR1-like 1    394 NAFVKLS-EDMVCLSMVP-TT-EVAIYGNFAQMDFLVGYDLETRTVSFQHMDCSANL
At CDR1-like 2    392 NAFVKLN-EDTVCLSMIP-TT-EVAIYGNMVQMDFLVGYDLETKTVSFQRMDCSGNL
At CDR1-like 3    379 NTFVAVS-EDVSCFAFAA-NEQ-LTIFGNLAQMNFLVGYDTVSGTVSFKKTDCSQM-
At CDR1           385 NAFVQVS-EDLVCFAFRG-SPS-FSIYGNVAQMNFLVGYDTVSKTVSFKPTDCAKM-
Pt GENMOD         361 NTFVQVS-DDVVCLAFAS-TTSGISIYGNVAQMNFLVEYNIQGKSLSFKPTDCT---
Vv CDR1-like 1    388 NTFVQVQ-EDLFCFAMIP-V-SDLAIFGNLAQMNFLVGYDLKSRTVSFKPTDCTKID
Vv CDR1-like 2    393 NTFVQAQ-EDLVCFSMIP-S-SNLAIFGNLSQMNFLVGYDLKNNKVSFKPTDCTKQ-
```
