# Supplementary figures and images for: Nodulin 41, a novel late nodulin of common bean with peptidase activity
Source: BMC Plant Biol. 2011 Oct 10;11:134. doi: 10.1186/1471-2229-11-134 (PMC3207901; doi:10.1186/1471-2229-11-134)

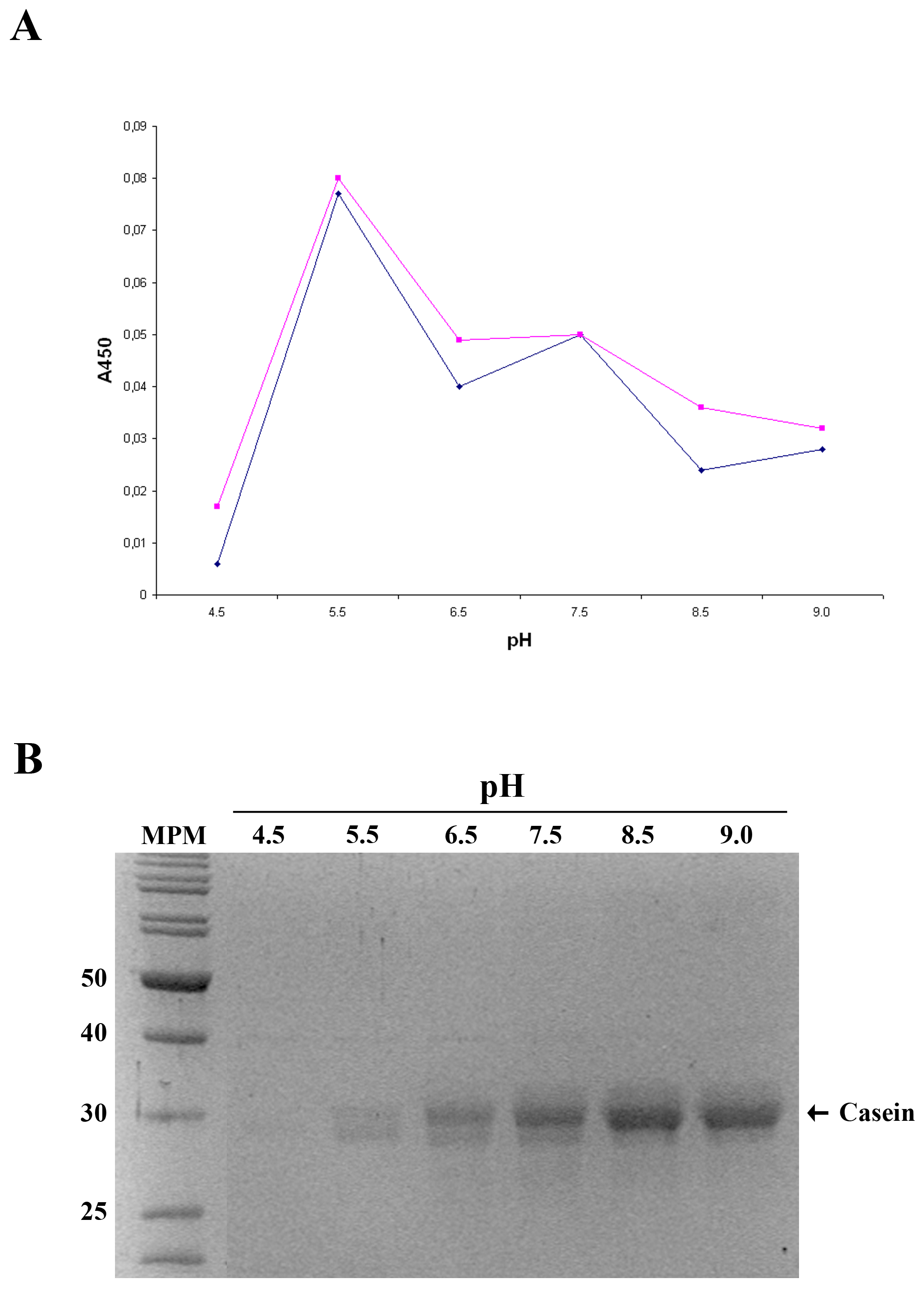

Supplement: Additional file 2 — PvNod41 peptidase activity detected with a chromogenic method. (A) Activity of PvNod41 on succinylated casein was assayed by using the QuantiCleaveTM Peptidase Assay kit (Pierce). Purified PvNod41 was incubated overnight at 37°C in different buffers at pH values between pH 4.5 and 9.0. The color produced by peptidase activity was measured at 450 nm and plotted against pH. Results of two independent experiments are shown. (B) Representative output of this assay on 12% SDS-PAGE analysis. The gel was stained with Coomassie Blue. [file 1471-2229-11-134-S2.TIFF]

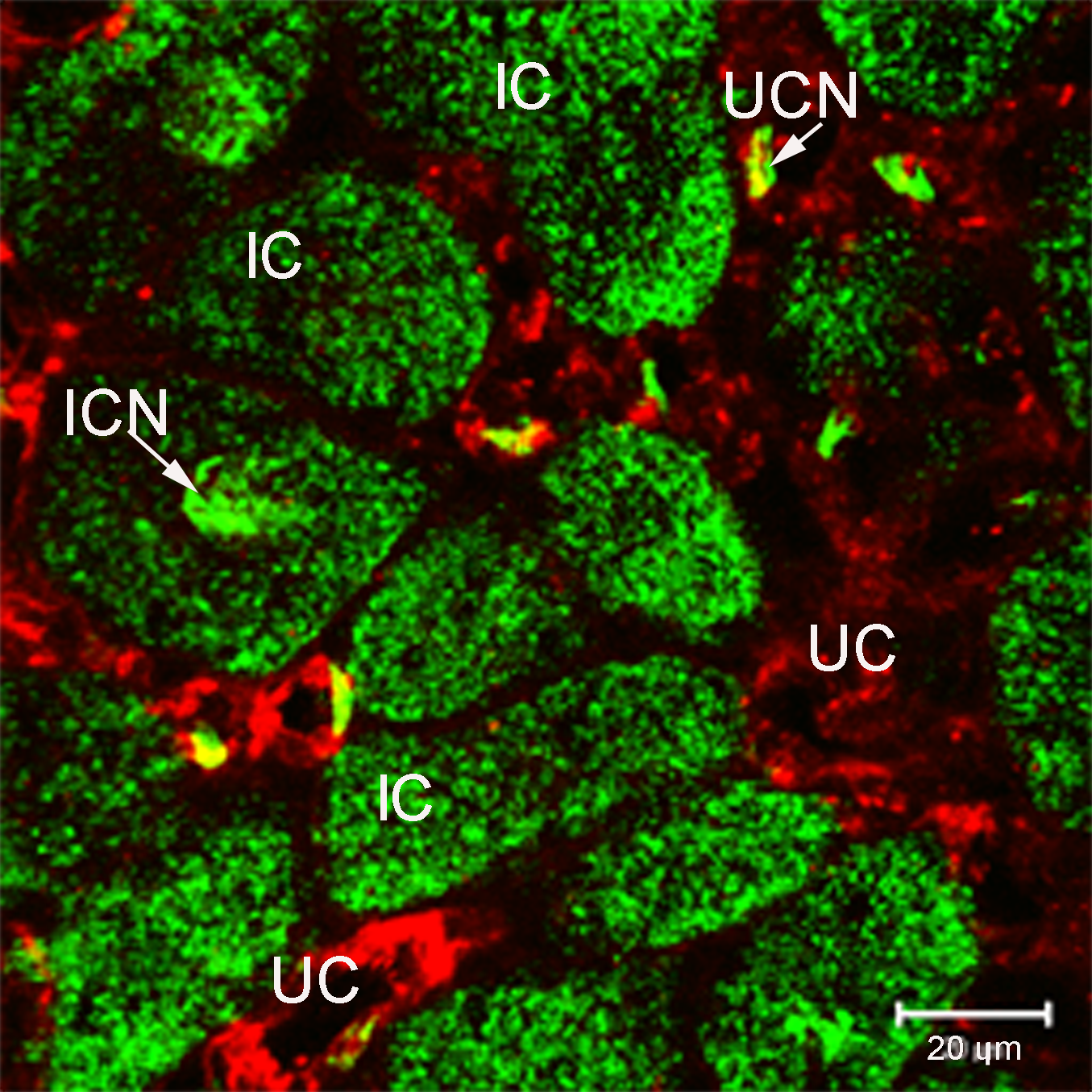

Supplement: Additional file 3 — Immunolocalization of PvNod41 in uninfected cells of common bean root nodule sections. PvNod41 was immunodetected with a specific antiserum. Anti-PvNod41 antibodies were visualized with a secondary antibody conjugated to Alexa Fluor® 633 (red), whereas bacteroids and nuclei were stained with Sytox Green (green). Uninfected cells (UC) containing PvNod41antigen can be clearly distinguished from infected cells (IC) containing bacteroids. ICN, Infected Cell Nucleus; UCN, uninfected cell nucleus. [file 1471-2229-11-134-S3.TIFF]
